# The Impact of Metformin on the Development of Hypothyroidism and Cardiotoxicity Induced by Cyclophosphamide, Methotrexate, and Fluorouracil in Rats

**DOI:** 10.3390/ph16091312

**Published:** 2023-09-16

**Authors:** Ahmad H. Alhowail, Maha A. Aldubayan

**Affiliations:** Department of Pharmacology and Toxicology, College of Pharmacy, Qassim University, Buraydah 51452, Saudi Arabia; m.aldubayan@qu.edu.sa

**Keywords:** cardiotoxicity, cyclophosphamide, fluorouracil, hypothyroidism, metformin, methotrexate, rats, thyroid hormones

## Abstract

Cyclophosphamide (CYP), methotrexate (MTX), and 5-fluorouracil (5-FU) are extensively utilized in the therapeutic management of various malignancies. It is noteworthy, however, that potential chemotherapy-related complications include the occurrence of hypothyroidism and cardiotoxicity. Metformin (MET) is a pharmacological agent for managing type 2 diabetes. It has been reported to mitigate certain toxic manifestations associated with chemotherapy. This study’s primary objective is to investigate MET’s protective effects against hypothyroidism and cardiotoxicity induced by CMF treatment. A total of forty male rats were allocated into four distinct groups, each consisting of ten rats per group. These groups were categorized as follows: saline, MET, CMF, and CMF + MET. The experimental group of rats were administered CMF via intraperitoneal injection, receiving two doses of CMF, and fed MET in their daily drinking water, with a 2.5 mg/mL concentration. Blood samples were collected into EDTA tubes for assessment of TSH, free and total (T4 and T3), troponin I, CK, and CK-MB levels utilizing Electrochemiluminescence Immunoassays (ECI). The saline and MET groups did not exhibit significant alterations in thyroid hormones or cardiotoxic biomarkers. In contrast, in the CMF group, there was a notable reduction in T4, FT4, T3, and FT3 levels but no significant changes in TSH levels; however, troponin I, CK, and CK-MB levels were notably elevated. MET co-treatment with CMF did not ameliorate these effects caused by CMF. In conclusion, CMF treatment induced hypothyroidism and cardiotoxicity in rats, but MET co-treatment did not rescue the reduction of thyroid hormones or the elevation of cardiotoxic biomarkers.

## 1. Introduction

Chemotherapy’s major procedure of action is generating cytotoxicity, which makes it effective against many different forms of cancer [1,2]. Cardiotoxicity, nephrotoxicity, hepatotoxicity, and hypothyroidism are only a few of the toxicities that can result from chemotherapy [3,4]. Unfortunately, no therapeutic options are available to reduce chemotherapy’s negative effects. Indeed, our prior research has established a correlation between increased concentrations of pro-inflammatory cytokines, such as interleukin-6 (IL-6) and interleukin-1 (IL-1), and the occurrence of neurotoxicity and cognitive impairment subsequent to prolonged exposure to CMF, which is a combination of cyclophosphamide (CYP), methotrexate (MTX), and 5-fluorouracil (5-FU) [5]. Therefore, this study investigated whether MET can alleviate the thyroid from CMF’s damaging effects by measuring thyroid hormone levels following treatment.

CMF is a triad of chemotherapeutic agents frequently employed in synergistic regimens for the management of diverse malignancies [6]. The effects of CYP are mediated via the process of DNA alkylation, leading to the inhibition of DNA synthesis and RNA transcription [7]. MTX, or methotrexate, is a pharmacological agent that acts as a folate antagonist [8]. It exerts its effects by inhibiting various enzymes involved in the synthesis of nucleotides [9]. The dihydrofolate reductase enzyme plays a crucial role in converting dihydrofolate to tetrahydrofolate, which plays a central role in facilitating the biosynthesis of DNA and RNA nucleotides [9,10]. In its capacity as an antimetabolite, 5-FU effectively hinders the process of thymidylate formation from uracil, thereby impeding the synthesis of DNA and RNA [11]. Moreover, numerous studies have demonstrated that the treatment with CYP, MTX, and 5-FU leads to a reduction in the generation of new neurons in the brain while also causing cardiotoxic effects [12,13,14]. These drugs’ adverse effects have been observed to contribute to cognitive impairment and diminished cardiac function [12,13,14]. Furthermore, a recent investigation involving breast cancer patients who underwent chemotherapy unveiled a notable manifestation of pronounced fatigue symptoms that were concomitant with a discernible decline in thyroid functionality [15]. Henceforth, it is imperative to conduct additional research to elucidate the intricate interplay between chemotherapy and thyroid function as well as hormone levels.

The thyroid gland is subject to stimulation by thyroid-stimulating hormone (TSH), which is secreted by the anterior pituitary gland [16]. This stimulation prompts the synthesis and subsequent release of thyroid hormones, specifically triiodothyronine (T3) and thyroxine (T4), from the thyroid follicular cells [17]. Thyroid hormones exist in two distinct forms, namely free and total thyroxine (T4) and free and total triiodothyronine (T3) [18]. These hormones play a crucial role in the regulation of numerous physiological functions, such as the regulation of metabolism, growth, and development, with a particular emphasis on their significant impact on the brain and heart [18,19]. Upon the release of thyroid hormones into the bloodstream, they exhibit the ability to engage in binding interactions with thyroid hormone receptors (THRs) that are widely distributed throughout various anatomical regions of the body, encompassing the brain, bone, heart, intestine, liver, kidney, and thyroid gland [20,21]. THRs are a class of nuclear receptors that exhibit two distinct isoforms: THRα and THRβ. These isoforms display differential distribution patterns across various tissues [22]. The observation that mice lacking the THR gene exhibit cognitive impairment provides persuasive evidence for the essential role of THR in the regulation of cerebral development [23]. Furthermore, it has been observed through neuroimaging studies that patients diagnosed with hypothyroidism exhibit notable alterations in both the structural and functional aspects of their brains [24]. Reports indicate that reduced thyroid hormone levels may lead to cardiac output alterations [25]. Hence, it is imperative to maintain the optimal range of thyroid hormone levels to function in other physiological systems properly.

Hypothyroidism may also have negative effects on other organs, including the brain and the heart, as neuroimaging studies have revealed alterations in the brain structure and function of hypothyroid patients [24,25]. Moreover, hypothyroidism is distinguished by abnormal levels of TSH and free and total T4 and T3 [26]. The mechanism and etiology of CMF-induced hypothyroidism and agents that can be used to protect patients from toxicity have not been reported in the current literature. Therefore, additional research is necessary to validate the relationship between CMF and hypothyroidism and to develop new compounds that can reduce the toxic effect of CMF on the thyroid gland and its function, possibly by enhancing thyroid hormone levels.

Furthermore, it is imperative to acknowledge that cardiotoxicity represents a significant health concern, as it engenders a compromised blood flow to the various anatomical regions of the body, thereby diminishing the supply of oxygenated blood to the remaining physiological systems [27]. Cardiotoxicity, a frequently observed adverse event associated with chemotherapy, encompasses the potential detrimental effects on the cardiovascular system [28,29]. Notably, medications such as doxorubicin and cyclophosphamide have been implicated in this phenomenon [30,31,32]. It is crucial to acknowledge that individuals with pre-existing cardiac impairments are particularly susceptible to heightened morbidity and mortality rates in the presence of cardiotoxicity [33,34]. The biomarkers troponin I, creatine kinase (CK), and creatine kinase-MB (CK-MB) exhibit elevated levels subsequent to myocardial infarction, cardiac toxicity, and injury [35,36]. Henceforth, these biomarkers serve as indicators of the manifestation of cardiotoxicity [36]. Multiple lines of compelling evidence have been documented, indicating that MET possesses the capacity to counteract chemotherapy-induced cardiomyopathy [37,38]. This is achieved through a reduction in elevated levels of troponin I and CK-MB, thereby leading to notable enhancements in cardiac outputs [37,39,40].

**Figure 1:**
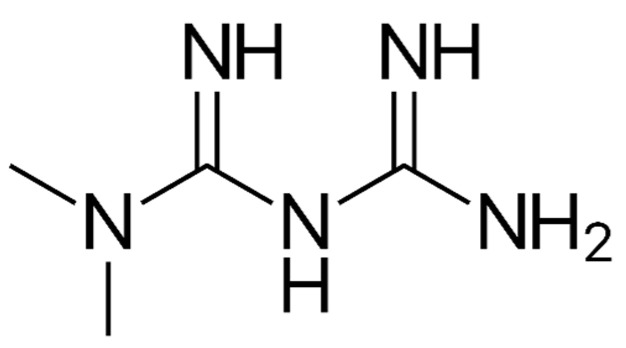
The chemical structure of metformin.

Metformin (MET) is a biguanide-class drug used to treat diabetes, and its chemical name is N, N-dimethylimidodicarbonimidic diamide (Figure 1). Inhibiting hepatic gluconeogenesis and increasing insulin receptor sensitivity are the primary mechanisms by which glucose levels are lowered [41]. MET has been used for decades as a successful treatment for diabetes mellitus [42]. It is the first-line treatment for diabetes mellitus and the most widely used oral antihyperglycemic medication [43]. In addition to its use in the treatment of diabetes, polycystic ovarian syndrome, cancer, and metabolic syndrome, metformin has been linked to a number of other health benefits [44,45]. Metformin’s ability to reduce the side effects of drugs like chemotherapy and increase patient survival has been supported by multiple lines of data [46,47].

Recent studies reported that MET can improve neurotoxicity and brain damage caused by chemotherapeutic agents such as cyclophosphamide and cisplatin in rodents [46,48]. In addition, our recent study also evaluated the neurotoxic effects induced by CMF therapy on the function of the brain, and we also studied the potentially ameliorative effect that may be achieved by combining MET and CMF. The results revealed that rats treated with CMF displayed signs of neurotoxicity, including increased neuroinflammation (which was demonstrated by increased levels of IL-1, tumor necrosis factor alpha, and IL-6), as well as increased levels of glutamate and dopamine in the brain [1,49]. The co-treatment with MET did not, however, have an ameliorating effect on these outcomes [1,49]. Furthermore, the hippocampal neurons were exposed to CMF, which resulted in a notable decline in the activity of mitochondrial complex I. In order to address these detrimental effects on mitochondrial function, MET was explored as a potential protective agent [50]. These data provide evidence that MET does not contribute to the favorable benefits of attenuating the neurotoxicity caused by CMF.

Consequently, the primary objective of this study is to examine the impact of CMF treatment on thyroid hormone levels and ascertain the potential mitigating effect of MET co-treatment in alleviating the deleterious consequences of CMF treatment.

## 2. Results

### 2.1. Effect of CMF and MET on Mortality

**Figure 2:**
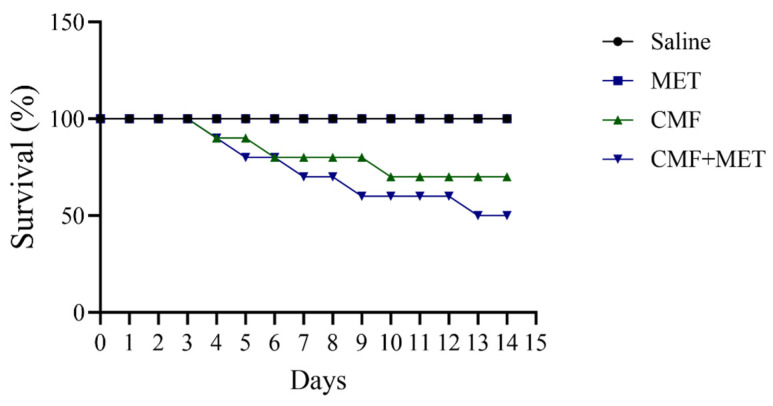
The influence of CMF and MET on the overall survival rate of rats.

The survival rate was unaffected by either saline or MET treatment. It is true that CMF treatment reduced rat survival by 40%; however, we observed a higher mortality rate in rats that were given both CMF and MET (Figure 2). After two weeks of treatment, 50% of the rats given CMF and MET died.

### 2.2. Effect of CMF and MET on the Body Weight

**Figure 3:**
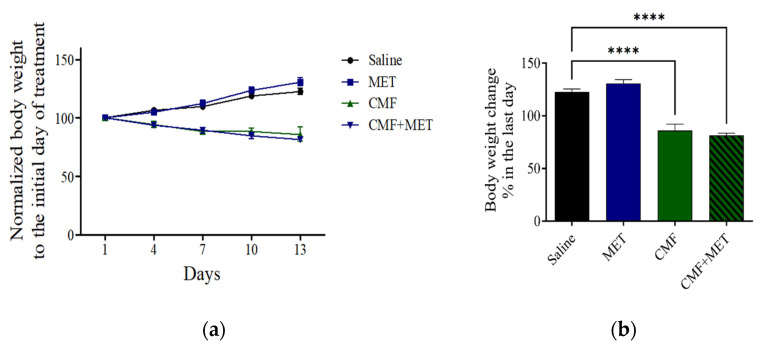
The potential influence of CMF and MET on the body weight of rats is investigated. (**a**) The observed alteration in the body weight throughout the course of the study. (**b**) The observed alteration in percentage on the final day, subsequent to the administration of therapeutic interventions. (**** *p* < 0.0001) related to saline rats.

The findings indicate that both the saline and MET groups exhibited a gradual increase in body weight. Nevertheless, it is noteworthy that a statistical decrease in body weight was observed in the group of rats administered with CMF and the group of rats administered with CMF + MET, when related to the group of rats receiving saline (Figure 3a,b).

### 2.3. The TSH Levels Were Unaffected by CMF or MET

**Figure 4:**
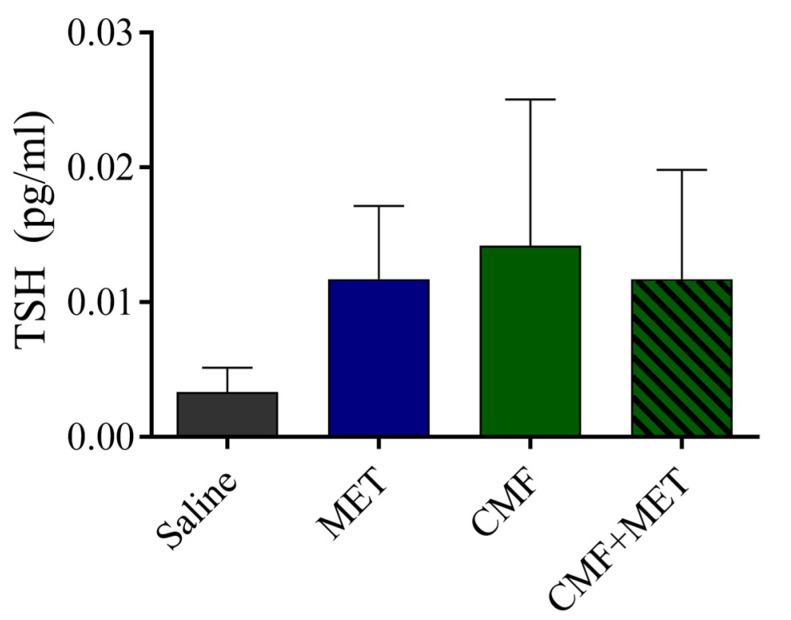
CMF, MET, and combination effects on TSH levels. The graph shows that there are no notable changes between the four studied rat groups. The findings are shown as SEM for seven animals in each group and were analyzed using one-way ANOVA.

The results of the electrochemiluminescence immunoassays (ECL) revealed no statistically significant differences in the concentrations of thyroid-stimulating hormone (TSH) among the experimental groups, namely the saline, CMF, MET, and CMF + MET groups. Nevertheless, a marginal elevation in the CMF and CMF + MET cohorts exhibited potential alterations in the thyroid-stimulating hormone (TSH) levels. Consequently, both the CMF and MET therapies exhibited negligible efficacy (Figure 4).

### 2.4. MET Did Not Reduce CMF Toxic Effects on T4 and FT4 Levels

**Figure 5:**
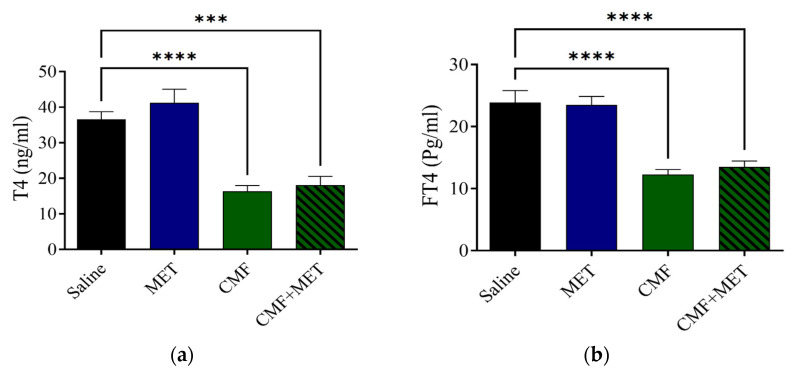
CMF, MET, and combination effects on T4 and FT4 levels. (**a**,**b**) CMF treatment (100 mg/kg CYP, 4 mg/kg MTX, and 100 mg/kg 5-FU, intraperitoneal injection) caused a significant decrease in the T4 and FT4 levels, but MET treatment did not affect the T4 and FT4 levels. Data are expressed as SEM for seven rats in each rat group. (*** *p* < 0.001) and (**** *p* < 0.0001) related to saline rats.

According to the findings of the ECL analysis, the levels of T4 and FT4 in the group that had been treated with CMF had a significantly reduced concentration when compared with the rats that had been given saline. The T4 and FT4 levels did not improve despite the use of co-treatment with MET (Figure 5a,b).

### 2.5. MET Did Not Improve CMF Toxic Effects on T3 and FT3 Levels

**Figure 6:**
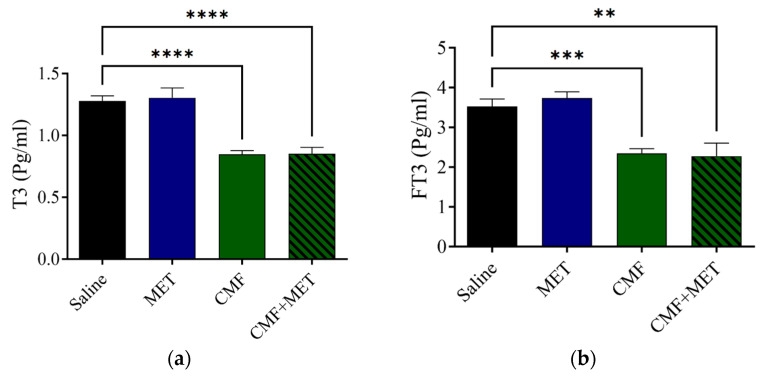
CMF, MET, and combination influence T3 and FT3 levels. (**a**,**b**) CMF treatment (100 mg/kg CYP, 4 mg/kg MTX, and 100 mg/kg 5-FU, intraperitoneal injection) significantly decreased the T3 and FT3 levels related to the saline group, but MET treatment did not alter the T3 and FT3 levels. CMF + MET co-treatment showed no positive effect on T3 and FT3 levels. The expression of data is given as SEM for seven rats in each rat group. (** *p* < 0.01), (*** *p* < 0.001), and (**** *p* < 0.0001) related to the saline rats.

The ECL analysis exhibited that the levels of T3 and FT3 were notably lower in concentration in the CMF group in comparison with the saline rats; however, the MET therapy on its own did not appear to have a significant impact on the T3 or FT3 levels in comparison with the saline group. The combination of MET and CMF did not appear to have any effect (Figure 6a,b).

### 2.6. Effect of CMF and MET on Troponin I, CK, and CK-MB Levels

**Figure 7:**
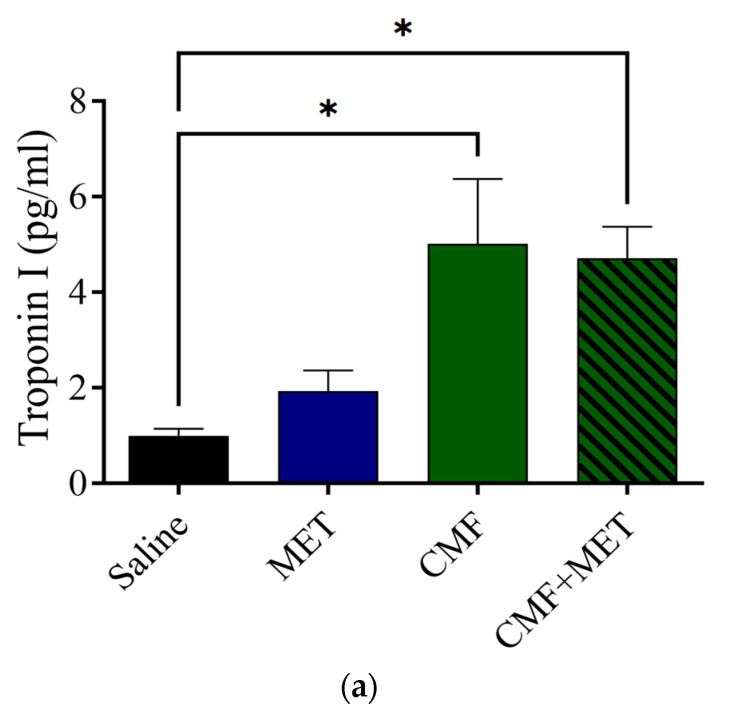

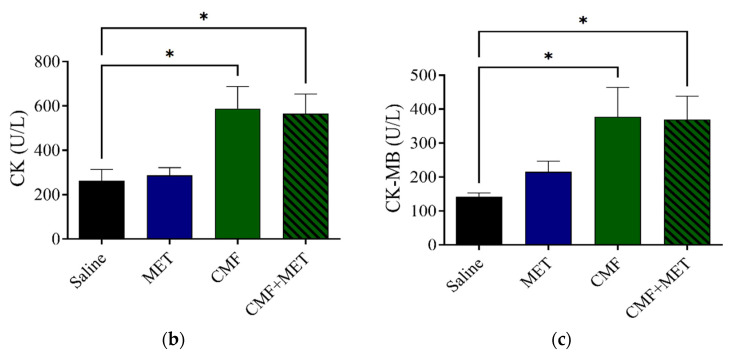
Effects of CMF, MET, and combination on troponin I, CK, and CK-MB levels. (**a**–**c**) CMF treatments (100 mg/kg cyclophosphamide, 4 mg/kg methotrexate, and 100 mg/kg fluorouracil, intraperitoneal injection) led to a noticeable elevation in troponin I, CK and CK-MB levels related to the saline group. MET treatment did not alter troponin I, CK, and CK-MB levels; however, CMF + MET co-treatment did not rescue the elevation of troponin I, CK, and CK-MB levels caused by CMF treatment. The expression of data was as SEM for seven rats in each rat group. (* *p* < 0.05) related to the control rats.

The findings indicate that the MET group exhibited no changes in the troponin I, CK, and CK-MB levels related to the saline group. However, the CMF- and CMF + MET-treated rat groups revealed a significant increase in troponin I, CK, and CK-MB levels related to the saline group (Figure 7a–c).

## 3. Discussion

In the present investigation, we conducted an assessment to determine the influence of CMF intervention on the physiological functions of the thyroid and cardiac systems in rat models. This was achieved by evaluating the concentrations of thyroid-stimulating hormone (TSH), thyroxine (T4), free-thyroxine (FT4), triiodothyronine (T3), and free-triiodothyronine (FT3) in addition to troponin I, CK, and CK-MB levels in plasma samples. Furthermore, this study postulated that the concurrent administration of MET and CMF may potentially confer a safeguarding effect against the deleterious impact of CMF on the thyroid gland and cardiac tissue. Nevertheless, the evidence presented thus far indicates that MET does not elicit any discernible modifications in thyroid or cardiac functions, whether independently or in the presence of hypothyroidism or cardiotoxicity in rats. Consequently, it can be inferred that MET does not possess any protective effects on the aforementioned physiological functions of the thyroid or heart.

Furthermore, it is noteworthy to mention that the groups administered saline and MET in isolation did not exhibit any discernible impact on the overall survival rate during the entire duration of the study. Nevertheless, it is worth noting that within the group subjected to CMF treatment, there was an observed occurrence of mortality, leading to a notable decrease in the overall survival rate, which amounted to 40 percent upon the conclusion of the study’s duration. Furthermore, a heightened incidence of mortality was noted in rodents subjected to the concomitant administration of CMF and MET, with a duration of only 50 percent throughout the duration of the investigation. Therefore, the simultaneous administration of MET in conjunction with CMF has the potential to induce increased drug toxicity or elicit a synergistic response against cellular entities, ultimately leading to cellular demise. The aforementioned observation displays similarities to our previous findings, wherein the rodents exposed to CMF demonstrated significantly increased concentrations of glutamate and dopamine, accompanied by the occurrence of mitochondrial dysfunction and lipid peroxidation in the brains of both the CMF- and CMF + MET-treated group. These alterations ultimately result in neuronal impairments and subsequent demise [1,51]. Moreover, it is important to highlight that the administration of CMF treatment led to the manifestation of adverse reactions. The observed augmentation in body weight reduction was apparent in both the CMF and CMF + MET cohorts, in comparison with the saline and MET monotherapy cohorts. The observed outcome pertaining to the body weight in rats exhibited a notable decrease subsequent to a duration of two weeks of administration with CMF and CMF + MET.

Based on the available clinical and experimental research evidence, it has been observed that chemotherapy has the potential to exert an influence on thyroid function [52,53]. Hence, the research outcomes have demonstrated that the administration of CMF exhibits a notable decrease in the concentrations of thyroid hormones, specifically T4, FT4, T3, and FT3 levels. These findings are in alignment with the initial observations reported in the study [3]. Thyroid hormone receptors exhibit significant expression in both the cerebral and cardiac tissues. However, a reduction in thyroid hormone levels can induce modifications in receptor functioning, thereby potentially impacting the physiological activities of the heart and brain [54,55]. Hypothyroidism has been associated with cardiac dysfunction and deficiencies in the brain, which can subsequently result in cognitive impairments [24]. Given the potential correlation between chemotherapy and hypothyroidism [3], further examination is necessary to understand the underlying mechanisms by which CMF affects thyroid function. This will help in identifying potential therapeutic interventions that can mitigate the negative effects of this association.

The primary aim of this research was to elucidate the pharmacological impacts of CMF, MET, and the combined administration of CMF with MET on the regulation of thyroid-stimulating hormone (TSH), thyroxine (T4), free thyroxine (FT4), triiodothyronine (T3), and free triiodothyronine (FT3) concentrations. The TSH levels were not found to have any notable changes following the administration of CMF or MET in any of the experimental groups, thus yielding an unexpected result. Rats subjected to CMF treatment displayed a notable reduction in T4, FT4, T3, and FT3 concentrations, while administration of MET alone did not elicit any observable changes in these variables. Similarly, no discernible changes were noted in the serum levels of T4, T3, FT4, or FT3 following the administration of MET co-treatment. Therefore, it has been noted that the utilization of MET has not exhibited effectiveness in reinstating individuals with hypothyroidism to the ideal levels of T4, T3, FT4, or FT3. Additional research is required to evaluate different dosages and durations of MET with the aim of mitigating the deleterious effects of CMF-induced hypothyroidism.

The previous literature has extensively reported that the administration of cyclophosphamide and fluorouracil can induce cardiotoxic effects, as indicated by the observed increase in troponin I and CK-MB concentrations [56,57,58]. The current study evaluated the concentrations of troponin I, creatine kinase (CK), and creatine kinase-MB (CK-MB) following CMF interventions. These biomarkers have been duly recognized as indicators of cardiotoxicity [35,36]. Henceforth, the findings have demonstrated a notable elevation in troponin I, CK, and CK-MB concentrations in comparison with the control group administered with saline. Moreover, it has been observed that MET demonstrated inefficacy in mitigating the deleterious effects on cardiac function, as indicated by the lack of any improvement in the concentrations of troponin I, CK, and CK-MB induced by CMF administration in rats. The observed pharmacological effects of MET appear to be in discordance with the reported research outcomes, implying that MET may potentially alleviate the cardiotoxic effects induced by cyclophosphamide or fluorouracil agents when administered as standalone treatments [40,59,60]. Numerous investigations have elucidated the promising capacity of MET to alleviate the cardiotoxic repercussions elicited by chemotherapeutic agents, such as doxorubicin, cyclophosphamide, and paclitaxel [59,61,62,63]. However, the results of this study suggest a dearth of amelioration in the cardiotoxic effects elicited by CMF treatment.

The current investigation possesses certain limitations attributable to its in vivo characteristics. Further in vitro investigations and subsequent clinical trials are warranted to validate and substantiate the findings. Moreover, our research was regrettably unable to delve into the intricate mechanisms underlying the impacts of CMF and MET on the intricate workings of thyroid and cardiac functions. The investigation of the potential impact of CMF on thyroid and cardiac functions, specifically on the thyroid gland and the heart tissues, holds considerable intrigue.

## 4. Materials and Methods

### 4.1. Study Area

The research was performed at Qassim University’s College of Pharmacy in the Kingdom of Saudi Arabia between 1 and 30 July 2021.

### 4.2. Drugs

Cyclophosphamide (CYP) was procured from Baxter (Mumbai, Maharashtra, India), Methotrexate (MTX) was acquired from Hospira UK Ltd. (Leeds, UK), Fluorouracil (5-FU) was sourced from Korea Company (Seoul, Republic of Korea), and Metformin hydrochloride (MET) was gained from Tabuk Pharma Company (Tabuk, Saudi Arabia).

### 4.3. Experimental Design

The animal house at Qassim University’s College of Pharmacy provided forty albino male rats weighing between 230 and 280 g for this study. The rodents spent their days in cages with a light-dark cycle of 12 h and a temperature of 25 ± 2 °C. The availability of food and water was never limited for them. The rodents were separated into four cohorts comprising ten rats each, wherein one cohort was designated as the control group receiving saline, another cohort received MET, the third cohort received CMF, and the fourth cohort received CMF + MET. Metformin (MET) was administered in the form of an aqueous solution, which was prepared by dissolving it in drinking water. The concentration of the solution was maintained at 2.5 mg per milliliter throughout the administration period [49]. The CMF was given intraperitoneally (i.p.) into rats (two doses of 50 mg/kg cyclophosphamide, 2 mg/kg methotrexate, and 50 mg/kg fluorouracil, spaced out over the course of two weeks) [49]. However, the MET in the CMF + MET group was given as a solution that was dissolved in drinking water at a concentration of 2.5 mg/mL following the initial CMF injection. The rats that were part of the control group were given two injections of saline. Following the completion of the daily observation of the rats’ mortality rate and body weight following treatments, they were subjected to biochemical assessment.

### 4.4. Electrochemiluminescence Immunoassays

On the 14th day, subsequent to the final administration of the drug, the rats were humanely euthanized through cervical decapitation following the induction of anesthesia using carbon dioxide (CO_2_). Blood specimens were expeditiously obtained and placed into tubes containing ethylenediaminetetraacetic acid (EDTA) from both the control group and the groups receiving treatment with MET, CMF, and CMF + MET. Subsequently, the vials containing the blood samples were subjected to centrifugation at a force of 12,000 times the acceleration due to gravity (12,000× *g*) for a duration of 10 min. The plasma, which underwent separation, was subsequently transferred to vials with a volume of 2 mL and stored at a temperature of −80 °C. Following the collection of blood samples, a comprehensive analysis was conducted employing a state-of-the-art fully automated analyzer. This sophisticated instrument employs a proprietary electrochemiluminescence technology to perform immunoassay analysis on various thyroid-related hormones, including TSH, T4, T3, FT4, and FT3, and cardiotoxicity biomarkers including troponin I, CK, and CM-MB. The ECL methodology was implemented utilizing the COBAS INTEGRA 400 plus system in strict accordance with the guidelines provided by the manufacturer, Roche Diagnostics, Germany.

### 4.5. Statistical Analysis

The data pertaining to the biomarkers associated with thyroid and cardiac function from the four groups (saline, MET, CMF, and CMF + MET) were subjected to unidirectional analysis of variance (ANOVA), and the results were presented as the mean, standard error, as calculated using the GraphPad Prism 10.0.0.153 software (GraphPad, Boston, MA, USA). The data were subjected to the Dunnett multiple comparison test to evaluate the endpoints against the control, and *p* 0.05 was counted as statistically significant.

## 5. Conclusions

The present investigation assessed the toxicological impact of CMF and explored the potential safeguarding effect of MET by employing rat models. The findings of this study offer substantiation indicating that CMF therapy may exert potential toxicological implications, as evidenced by an elevated mortality rate and a decrease in body weight. Additionally, it is plausible that this may result in the manifestation of hypothyroidism by means of diminishing levels of T4, FT4, T3, and FT3, without affecting TSH levels. Moreover, the results additionally revealed the potential occurrence of cardiotoxicity by means of heightened levels of troponin I, CK, and CK-MB. Regrettably, the aforementioned findings also suggest that MET does not bestow any protective properties against the toxic manifestations of hypothyroidism and cardiotoxicity induced by CMF in rat models.

## Data Availability

The pertinent data that substantiate the conclusions of this study can be gained from the corresponding author upon a reasonable inquiry.
